# Preparation of Coupling Catalyst HamZIF-90@Pd@CALB with Tunable Hollow Structure for Efficient Dynamic Kinetic Resolution of 1-Phenylethylamine

**DOI:** 10.3390/molecules28030922

**Published:** 2023-01-17

**Authors:** Pengcheng Li, Jingjing Zhu, Hongyu Zhang, Lan Wang, Shulin Wang, Mengting Zhang, Jianping Wu, Lirong Yang, Gang Xu

**Affiliations:** College of Chemical and Biological Engineering, Zhejiang University, Hangzhou 310058, China

**Keywords:** hollow amorphous, HamZIF-90@Pd@CALB, DKR, 1-phenylethylamine

## Abstract

Chiral amines are essential components for many pharmaceuticals and agrochemicals. However, the difficulty in obtaining enantiomerically pure amines limits their application. In this study, hollow amorphous ZIF-90 (HamZIF-90) materials were prepared by template engraving, and chemical–enzyme coupling catalysts (HamZIF-90@Pd@CALB) were constructed for the chiral resolution of 1-phenylethylamine. Different from conventional materials, HamZIF-90 had tunable hollow structures by altering its central node zinc ion concentrations, and the embedded hydrogel template gave it more pore structures, which facilitated the loading of enzyme molecules and Pd nanoparticles (NPs). The establishment of the coupling catalysts shortened the mass transfer distance of the reactant molecules between the metal nanoparticles and the enzyme catalyst in the dynamic kinetic resolution (DKR) reaction, resulting in 98% conversion of 1-phenylethylamine and 93% selectivity of Sel.R-amide. The proposal of this idea provided a good idea for future tailor-made MOFs loaded with chemical and enzyme coupled catalyst.

## 1. Introduction

Chiral amines are important components of pharmaceuticals, fragrances, and agrochemicals [1,2,3]. Dynamic kinetic resolution (DKR) is an effective means to obtain enantiomeric pure amines, which breaks the limitation of only 50% theoretical yield of enantiomers required in enzymatic reaction alone and can reach 100% theoretical yield after in situ combination with racemic process [4,5]. However, designing a mature DKR system is not straightforward. Racemic catalysts require high temperatures to destroy undesirable coordination sites and imine intermediates to facilitate the reaction, and this inevitably limits the performance of the enzyme [5,6]. Therefore, it is crucial to develop enzyme-compatible racemic schemes to achieve high selectivity for DKR reactions. Metal nanoparticles (MNPs) are the major racemic catalysts, which are prone to migration and agglomeration due to their own high surface energy [7]. To overcome this limitation, researchers have prepared loaded catalysts with well-dispersed and small metal particle sizes by using carriers [8,9]. Moreover, the enzyme has a considerable performance enhancement by immobilization. Zdeněk Sofer et al. [10] found that the tunable presence and distribution of hydrophobic domains with surrounding hydrophilic groups on the graphene oxide surface supported the effective binding to lipase and facilitated the thermal stability. The adsorption of lipase was accompanied by slight non-specific interactions that contributed to its maintenance in organic solvents. The studies by Omar K. Farha [11] and Abdol-Khalegh Bordbar [12] et al. similarly demonstrated that the immobilization of the enzyme significantly could enhance its stabilities. It can be seen that the immobilization strategy can take into account both the enzyme stability and the increased activity of the racemic catalyst and enhance the compatibility between them. Currently, various carriers such as magnetic nanoparticles (MN) [13], siliceous mesocellular foams (MCFs) [14,15], and metal-organic frameworks (MOFs) [16] have been used for the immobilization of DKR catalysts and obtained considerable enantioselectivity of pure amines under mild conditions. Meanwhile, the one-pot DKR reaction with two catalysts co-immobilized onto the one carrier has been shown to significantly improve the efficiency of the reaction [14]. However, current studies to solve the compatibility problem of pure amine DKR catalysts are mainly focused on the selection of different carriers, and fewer studies have been conducted to explore the influence of the porous structure of the carriers and the suitability between carrier cavity size and enzyme size in the reaction. Therefore, we performed a study in this aspect by utilizing the structural properties of MOFs.

MOFs are 3D networked crystalline porous structures composed of metal nodes and organic ligands with intrinsic properties such as high specific surface area, excellent chemical stability, easy post-modification, and tunable functional groups, making them ideal platforms for immobilization of enzymes and metals [17,18,19,20]. MNPs [16,21], enzymes [22,23,24], and pharmaceutical molecules [25] can be encapsulated in MOFs by impregnation, cross-linking/embedding, etc. Nevertheless, the mismatch between the pore size of MOFs and the size of enzymes affects the loading efficiency of enzymes because of, for example, leaching of enzymes from the MOFs matrix [26]. The long-distance transport of the substrate between MNPs and the enzyme also affects the reaction rate. Hence, it is necessary to design the desired structure of the MOFs by changing the metal ion concentration and adding template agents to increase the suitability between the enzyme and the carrier to improve the reaction efficiency [26,27].

**Scheme 1:**
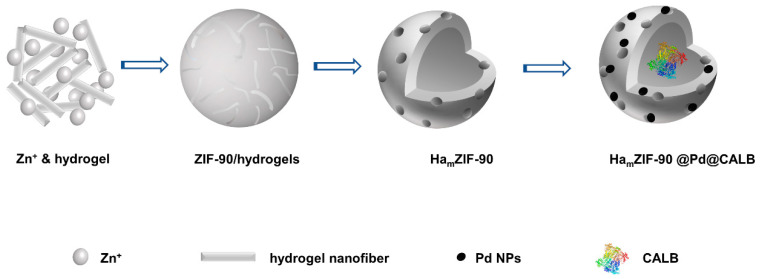
Schematic diagram of the synthesis process of hollow core-shell Ha_m_ZIF-90@Pd@CALB.

In this paper, Pd was chosen for its good racemization [4,16,28], and Candida antarctica lipase B (CALB) was selected for immobilization because of its high stability to organic solvents and elevated temperatures and its ability to catalyze a wide range of reactions including transesterification reactions [29,30,31]. The hollow amorphous material HamZIF-90 was prepared by using hydrogels engraved in the ZIF-90 framework [32]. The size of the cavity structure was adjusted by altering the concentration of the central node ions for enzyme encapsulation. The activity of the racemic catalysts was improved by preparing small-sized Pd NPs. Subsequently, Pd NPs and lipase CALB were co-immobilized on HamZIF-90 by adding stabilizers and cross-linkers, and the characterization results demonstrated the successful construction of the HamZIF-90@Pd@CALB catalyst; the preparation process is shown in Scheme 1. Finally, the DKR reaction was carried out with 1-phenylethylamine as the substrate and HamZIF-90@Pd@CALB as the catalyst and compared with the Novozym 435 commercial catalyst. HamZIF-90(0.06)@Pd@CalB showed good conversion (98%) and selectivity (Sel.R-amide 93%) under mild conditions.

## 2. Results and Discussions

### 2.1. Characterization of the As-Prepared Materials

**Figure 1:**
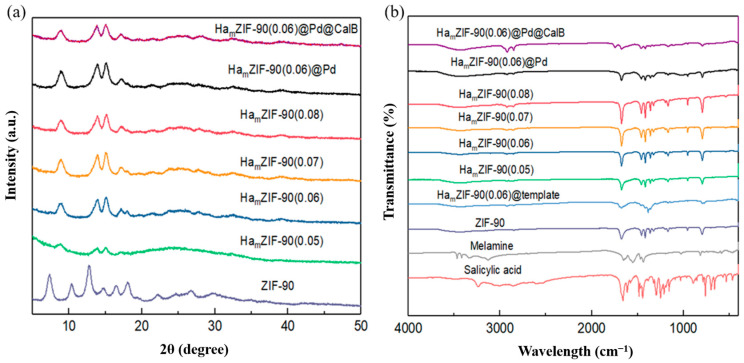
(**a**) XRD patterns and (**b**) FTIR patterns of salicylic acid, melamine, ZIF-90, Ha_m_ZIF-90(0.06)@template, Ha_m_ZIF-90(0.05), Ha_m_ZIF-90(0.06), Ha_m_ZIF-90(0.07), Ha_m_ZIF-90(0.08), Ha_m_ZIF-90@Pd, and Ha_m_ZIF-90@Pd@CALB.

Comparing the X-ray diffraction (XRD) patterns of ZIF-90 and Ha_m_ZIF-90, the XRD pattern of Ha_m_ZIF-90 shown in Figure 1a confirmed that the product had a random network topology [33] rather than the square sodimuite topology of the crystal ZIF-90 (CCDC No. 693596) [34]. Due to its amorphous nature, the product will be referred to as Ha_m_ZIF-90 in the following. The characteristic peaks of Ha_m_ZIF-90 still existed in the XRD patterns of Ha_m_ZIF-90@Pd and Ha_m_ZIF-90@Pd@CALB after the loading of Pd nanoparticles and CALB, which proved that the loading of Pd nanoparticles and CALB did not cause obvious damage to the structure of HamZIF-90, and the structure was stable.

The Fourier transform infrared (FT-IR) spectra of Ha_m_ZIF-90 and conventional ZIF-90 were observed as shown in Figure 1b. The characteristic peak at 1675 cm^−1^ was attributed to -CHO. The broad peak at 3480 cm^−1^ was attributed to free –OH stretching vibrations, which may have been caused by the physical adsorption of water in the air [35]. Notably, there was a characteristic peak belonging to Zn-N stretching that was observed at 534 cm^−1^, which indicated that the framework of ZIF-90 had been successfully formed. Compared with Ha_m_ZIF-90, Ha_m_ZIF-90 containing the gel template showed weaker stretching vibration peaks at 1000–1750 cm^−1^, but characteristic peaks still existed. It indicated that the characteristic groups were stable after removing the gel template. Furthermore, the NH_2_ in melamine led to a stretching vibration peak at 3469 cm^−1^ in the gel template contained in HamZIF-90. The phenolic hydroxyl group in salicylic acid had a characteristic peak at 3398 cm^−1^ [32]. In the FT-IR spectrum of Ha_m_ZIF-90, the disappearance of the characteristic peaks at 3469 cm^−1^ and 3398 cm^−1^ confirmed the removal of the gel template. Comparing the FT-IR spectra before and after loading the Pd nanoparticles and CALB, the characteristic peaks of the group of Ha_m_ZIF-90 loaded with Pd did not change. The peaks at 1675 cm^−1^ and 1293 cm^−1^ in Ha_m_ZIF-90@Pd@CALB were from the C=O bond of the peptide group and the C–N single bond vibration in the enzyme. All of these indicated the formation of the HamZIF-90 framework structure.

**Figure 2:**
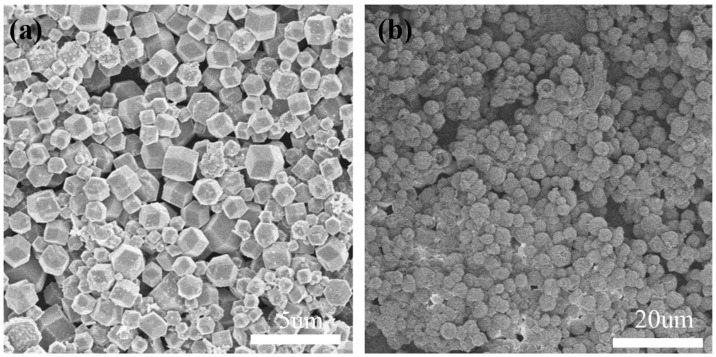
SEM images of (**a**) ZIF-90 and (**b**) HamZIF-90(0.06).

**Figure 3:**
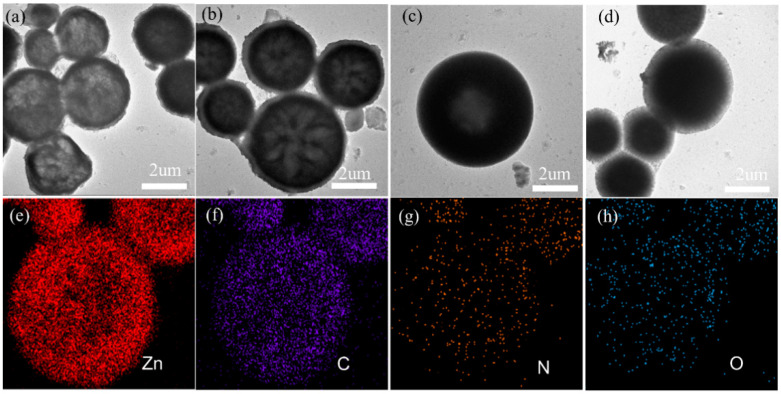
TEM images of (**a**) Ha_m_ZIF-90(0.05), (**b**) Ha_m_ZIF-90(0.06), (**c**) Ha_m_ZIF-90(0.07), and (**d**) Ha_m_ZIF-90(0.08). (**e**–**h**) EDS images of Zn, C, N, and O elements of Ha_m_ZIF-90(0.06).

The scanning electron microscope (SEM) images of ZIF-90 and Ha_m_ZIF-90 are shown in Figure 2, where it can be observed that the crystals of ZIF-90 had a regular and uniform dodecahedral shape with the particle size ranging from 1 to 3 μm. Ha_m_ZIF-90 had a hollow spherical shape with a particle size of about 3 μm and a rough surface. The morphology of Ha_m_ZIF-90 was further observed by transmission electron microscope (TEM), and the materials had a hollow structure with a rough surface as shown in Figure 3. The size of the cavity was regulated by controlling the concentration of Zn ions. It can be observed from Figure 3a–d sequentially that the cavity gradually became smaller as the concentration of Zn ions increased. The energy-dispersive X-ray spectroscopy (EDS) maps of HamZIF-90(0.06) characterized the elemental distribution of Zn, C, N, and O as shown in Figure 3e–h, with a distinct hollow morphology. After the hydrogel was engraved, the elemental composition of the central part was also reduced.

**Figure 4:**
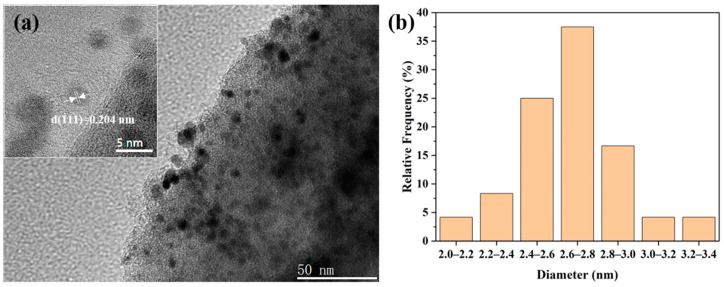
(**a**) HRTEM image and (**b**) diameter distribution of Pd NPs in Ha_m_ZIF-90(0.06)@Pd.

High-resolution TEM (HRTEM) studies suggested that the Pd NPs had a well-defined structure with sizes of 2–3 nm and were enclosed by {111} facets (Figure 4a,b) [21]. The actual Pd content of the composite was determined to be 2.244 wt.% by inductively coupled plasma atomic emission spectroscopy (ICP-AES).

**Figure 5:**
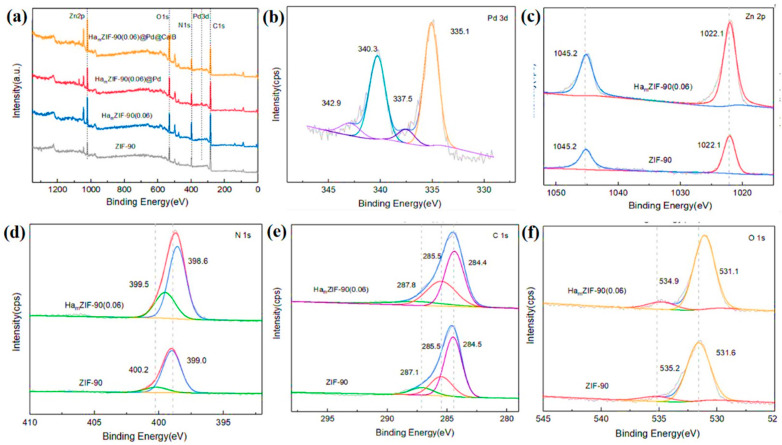
(**a**) XPS spectrum of ZIF-90, Ha_m_ZIF-90(0.06), Ha_m_ZIF-90(0.06)@Pd, and Ha_m_ZIF-90(0.06)@ Pd@CALB. (**b**) Pd 3d spectra of Ha_m_ZIF-90(0.06)@Pd. (**c**–**f**) ZIF-90 and Ha_m_ZIF-90(0.06) XPS spectrum of (**c**) Zn 2p, (**d**) N 1s, (**e**) C 1s, and (**f**) O 1s.

To demonstrate whether the elemental compositions of Ha_m_ZIF-90 and ZIF-90 were the same, the elemental spectra of the two were detected by X-ray photoelectron spectroscopy (XPS) as shown in Figure 5a. The results showed that Ha_m_ZIF-90 and ZIF-90 had the same the elemental compositions with peaks corresponding to Pd 3d, Zn 2p, O 1s, N 1s, and C 1s. Compared with Ha_m_ZIF-90(0.06), the peaks of Ha_m_ZIF-90(0.06)@Pd and Ha_m_ZIF-90(0.06)@Pd@CALB in the Zn 2p, O1s, N1s, and C1s regions were almost identical, which further indicated that Ha_m_ZIF-90 was stable during the loading processes of Pd NPs and CALB. The Pd 3d spectrum of Ha_m_ZIF-90(0.06)@Pd appeared at 300–400 eV as shown in Figure 5b. Two main peaks at 335.1 eV and 340.3 eV indicated the presence of Pd (0), and two small peaks at 337.5 eV and 342.9 eV indicated the presence of small amounts of Pd (II); thus, the Pd of Ha_m_ZIF-90(0.06)@Pd mainly existed in the singlet state.

According to the literature, the N 1S of the imine and secondary amine groups of imidazole-2-formaldehyde corresponded to peaks of 398.5 eV and 400.2 eV as shown in Figure 5c [33]. The peak at 399.0 eV in the spectra of N 1s of ZIF-90 corresponded well to the peak of tertiary amine, which was due to the disappearance of the imine group after coordination with Zn^2 +^. A small part of the secondary amine groups persisted because of the presence of uncoordinated ICA joints on the outer surface of the particles. The 398.6 eV and 399.5 eV peaks of N 1s of HamZIF-90(0.06) relative to the 399.0 eV and 400.2 eV peaks of ZIF-90 and the 531.1 eV and 534.9 eV peaks of O 1s (Figure 5f) of HamZIF-90(0.06) relative to the 531.6 eV and 535.2 eV peaks of ZIF-90 both were obviously displaced, which may have been attributed to the introduction of the hydrogel changing the electronic environment of the elements and promoting charge transfer. Further, Ha_m_ZIF-90(0.06) was almost the same as ZIF-90 in the XPS spectrum of Zn 2p (Figure 5c) and C 1s (Figure 5e).

**Figure 6:**
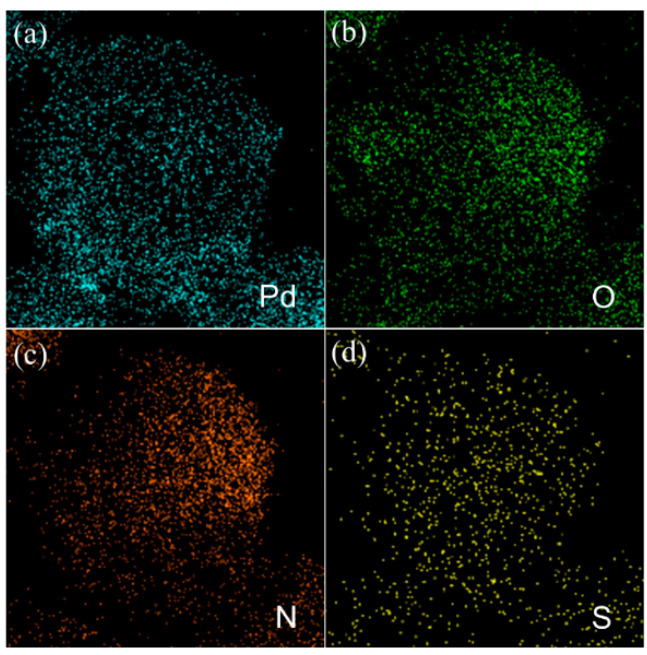
EDS images of (**a**) Pd, (**b**) O, (**c**) N, and (**d**) S elements of Ha_m_ZIF-90(0.06)@Pd@ CALB.

The EDS analysis spectrum of Ha_m_ZIF-90(0.06)@Pd@CALB is shown in Figure 6, which characterizes the distribution of Pd, O, N, and S elements. It can be observed that the Pd and S elements were evenly distributed on Ha_m_ZIF-90(0.06), indicating that Pd NPs and CALB had been successfully loaded on Ha_m_ZIF-90(0.06). It is worth noting that even after loading the enzyme and Pd NPs, Ha_m_ZIF-90(0.06) remained a good topology, indicating that the composite material had stability.

**Figure 7:**
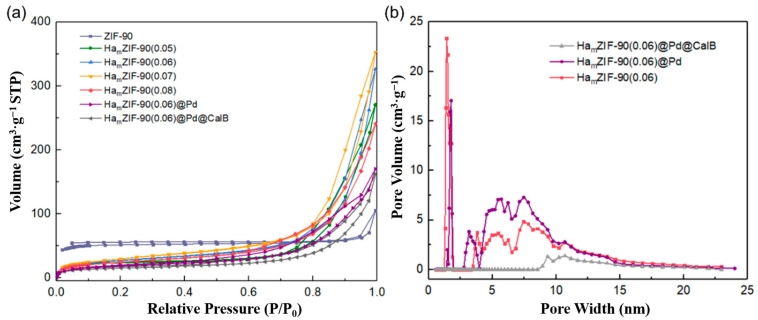
(**a**) N_2_ adsorption–desorption isotherms of Ha_m_ZIF-90, Ha_m_ZIF-90(0.06)@Pd, and Ha_m_ZIF-90(0.06)@Pd@CALB. (**b**) DFT image of Ha_m_ZIF-90(0.06), Ha_m_ZIF-90(0.06)@Pd, and Ha_m_ZIF-90(0.06)@Pd@CALB.

The specific surface area of Ha_m_ZIF-90 was measured and analyzed by the nitrogen adsorption–desorption curves as shown in Figure 7a. It could be seen that the isotherm trends for HamZIF-90(0.05), HamZIF-90(0.06), HamZIF-90(0.07), and HamZIF-90(0.08) were similar to the type III isotherm, indicating that HamZIF-90 has mesoporous and macroporous properties owing to the etching of the hydrogel by the high-temperature aqueous solution [36]. According to the BET model, the specific surface areas of Ha_m_ZIF-90(0.05), Ha_m_ZIF-90(0.06), Ha_m_ZIF-90(0.07), and Ha_m_ZIF-90(0.08) were 89 m^2^·g^−1^, 91 m^2^·g^−1^, 101 m^2^·g^−1^, and 110 m^2^·g^−1^, respectively, and the specific surface area of ZIF-90 was 204 m^2^·g^−1^. It can be seen that the specific surface area of HamZIF-90 gradually decreased with the decreasing of the Zn ion concentration, which confirmed the densification of Ha_m_ZIF-90 during the amorphous process [34].

Meanwhile, the specific surface area of Ha_m_ZIF-90(0.06)@Pd (69 m^2^·g^−1^) loaded with Pd NPs and Ha_m_ZIF-90(0.06)@Pd@CALB (56 m^2^·g^−1^) loaded with Pd NPs and CALB were smaller than that of Ha_m_ZIF-90(0.06) (91 m^2^·g^−1^), which was attributed to the fact that Pd NPs and CALB occupied part of the pores in the structure of Ha_m_ZIF-90(0.06). Furthermore, according to the NLDFT pore size distribution as shown in Figure 7b, the main pore size distributions of Ha_m_ZIF-90(0.06) and Ha_m_ZIF-90(0.06)@Pd ranged from 1.4 nm to 1.8 nm and 3.0 nm to 10.0 nm, which demonstrated that the loading of Pd NPs did not affect the main structure of Ha_m_ZIF-90(0.06). However, the Ha_m_ZIF-90(0.06)@Pd@CALB had no pore size in the range of less than 5 nm after immobilizing lipase CALB, which indicated that the loading of the enzyme occupied the micropores.

**Figure 8:**
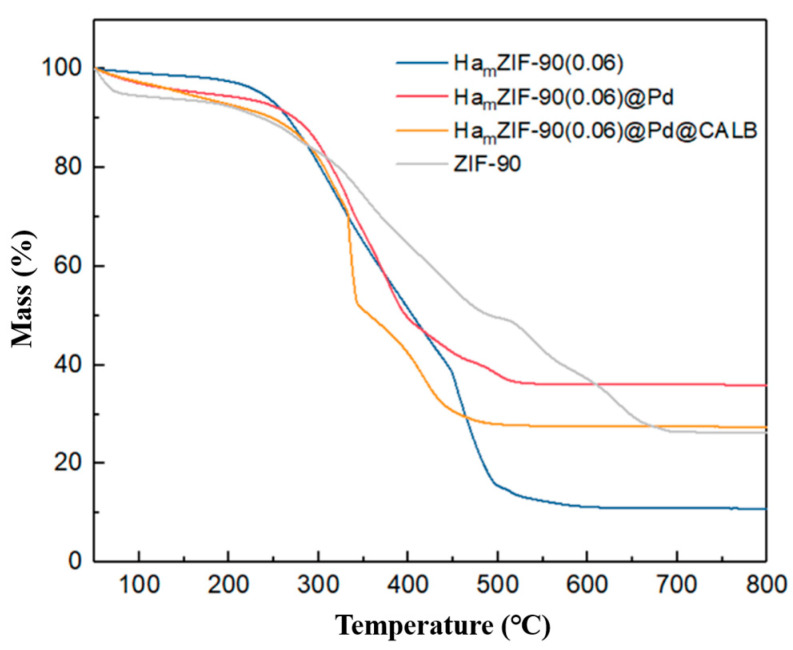
TGA curves of ZIF-90, Ha_m_ZIF-90(0.06), Ha_m_ZIF-90(0.06)@Pd, and Ha_m_ZIF-90(0.06)@Pd@CALB.

In order to study the thermal stability of the synthetic materials, its thermogravimetric analysis (TGA) curve is shown in Figure 8. In the range 50–200 °C, the mass loss was mainly due to the evaporation of adsorbed water and ethanol. In the range 200–450 °C, the solvent was further removed from the cavity structure, and the oxygen-containing (O) groups were decomposed. The weight loss of HamZIF-90 was significantly larger than that of ZIF-90 due to the decomposition of the carboxyl group of salicylic acid in the unremoved hydrogel. The weight loss at temperatures above 300 °C was also due to the progressive degradation of the ZIF framework. In the range 450–800 °C, the organic linker molecules in ZIF-90 decomposed further, and the final residue was the oxide (ZnO). Similarly, the Ha_m_ZIF-90 had essentially completed its decomposition in the range of 450–500 °C. The TGA curves of Ha_m_ZIF-90(0.06)@Pd and Ha_m_ZIF-90(0.06)@Pd @CALB showed similar patterns as mentioned above. The samples of Ha_m_ZIF-90(0.06)@Pd@CALB showed a deeper drop at 320 ℃, which was attributed to the decomposition of CALB with the occurrence of a weight loss of about 18% [37].

### 2.2. DKR Reaction of 1-Phenylethylamine

**Scheme 2:**
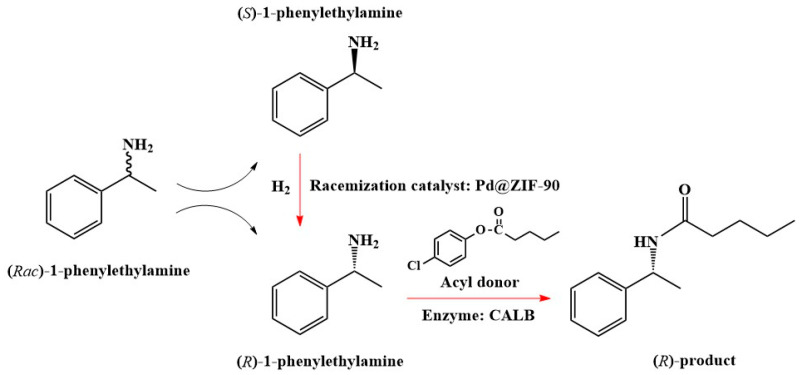
DKR reaction of 1-phenylethylamine.

During this reaction of DKR (shown in Scheme 2), lipase catalyzed the (R)-chiral amine and the acyl donor to generate the (R)-chiral amide, and the remaining (S)-chiral amine were continuously racemized under the action of chemical catalyst to obtain a single conformation enantiomer in a theoretical yield of 100%.

To investigate the catalytic performance of the racemization of HamZIF-90@Pd synthesized with different Zn ion concentrations, HamZIF-90@Pd was used as the racemization catalyst with (S)-1-phenylethylamine as the substrate and reacted at 60 °C and 0. 03 MPa hydrogen pressure for 12 h. As shown in Table 1, it can be seen from the results that the racemization performance of HamZIF-90@Pd on (S)-1-phenylethylamine was close to the racemization reaction, and HamZIF-90(0.06)@Pd performed relatively well with a conversion of 42% and ee_amine_ of 12%, while HamZIF-90 was not catalytically active when used alone.

Subsequently, one-pot DKR reaction with (*rac*)-1-phenylethylamine as substrate was carried out. Novozyme 435 is a well-established commercial heat-stable lipase used separately in combination with a synthetic chemical catalyst. The reaction was carried out with the HamZIF-90 series catalyst loaded with Pd NPs and CALB lipase, and the catalytic results are shown in Table 2, from which it can be seen that HamZIF-90(0.06)@Pd@CalB showed better conversion (98%) and selectivity (93% for Sel.R-amide) compared with the other catalysts.

HamZIF-90(0.06)@Pd@CALB showed a better catalytic effect than the combination of ZIF-90@Pd and Novozyme 435, probably due to the co-immobilization of Pd NPs and CALB on the same carrier, which shortened the mass transfer distance and accelerated the molecular transfer rate, resulting in a significant improvement in the conversion rate of the substrate and the selectivity of the target product. The higher catalytic activity of HamZIF-90@Pd@CALB compared with ZIF-90@Pd@CALB may be due to the hollow structure of HamZIF-90, which can shorten the diffusion distance, increase the mass transfer rate, and provide sufficient space for the phenylethylamine molecules to contact the surface active site, thus increasing the catalytic activity.

Racemization is a dynamic equilibrium process between dehydrogenation and hydrogenation, so hydrogen pressure is the key factor for successful racemization. Based on the good catalytic activity of HamZIF-90(0.06)@Pd@CALB as described above, this catalyst was chosen to explore the effect of DKR reactions at different pressures, and the results are shown in Table 3. Comparing the catalytic effect at different pressures, it was found that higher conversion and selectivity could be obtained at a hydrogen pressure of 0.03 MPa. At lower hydrogen pressures, the dehydrogenation reaction could be promoted, facilitating the formation of the target product and improving selectivity, while too low a hydrogen pressure slows down the racemization process and reduces the progress of the reaction.

Similarly, HamZIF-90(0.06)@Pd@CALB was chosen as the catalyst to explore the effect of different solvents on the DKR reaction, and the results are shown in Table 4. It can be found that the best conversion and selectivity were achieved in nonpolar solvent toluene, while those in the polar solvent were relatively low. This was attributed to the fact that in a nonpolar solvent environment, the less polar intermediate imine was more stable than the more polar substrate amine, which facilitated the racemization process.

## 3. Materials and Methods

### 3.1. Materials

Zinc nitrate hexahydrate [Zn(NO_3_)_2_·6H_2_O] (99%, AR), melamine (99%, AR), polyvinylpyrrolidone K30 (GR), N-hexane (AR), Dichloromethane (AR), sodium borohydride (>98%), anhydrous ethanol (99.7%, AR), sodium hydroxide (>96%, AR), isopropyl alcohol (99.7%, AR), and toluene (99.5%, AR) were purchased from Sinopharm Chemical Reagent Co., Ltd. (Shanghai, China). Salicylic acid (99.5%, AR), glutaric dialdehyde (AR), and CALB (immobilized lipase B from Candida Antarctica) were purchased from Macklin Biochemical Co., Ltd. (Shanghai, China). Span 85 (AR), Palladium(II) acetylacetonate (99%), rac-1-Phenylethylamine (99%), and (S)-1-Phenethylamine (>98%) were purchased from Aladdin Bio-Chem Technology Co., Ltd. (Shanghai, China). Imidazole-2-carbaldehyde (>95%) was purchased from Bide Pharmatech Ltd. (Shanghai, China). The 4-Chlorophenyl pentanoate was obtained via chemical organic synthesis. Hydrogen (>99.99%) was obtained from Hangzhou Minxing Chemical Technology Co., Ltd. (Hangzhou, China).

### 3.2. Characterization

The diffraction patterns of the catalysts were recorded by an X-ray diffractometer (XRD) (PANalytical X’Pert diffractometer) in the range of 2θ = 2–50°. The Fourier infrared spectrogram (FT-IR) patterns of the samples were measured by Fourier infrared spectrometer (SGE @Agilent 6890@Nicolet 5700, wavelength 4000–400 cm^−1^, 32 scans). The crystal morphologies of the catalysts were investigated by field emission scanning electron microscope (SU-8010-SEM (Hitachi, Japan), acceleration voltage 3 kV) and 120 kV transmission electron microscope (HT-7700-TEM (Hitachi, Japan)). In addition, high-resolution TEM (JEM-2100, acceleration voltage 200 kV) was used to observe lattice stripes on the surface of metal nanoparticles. Energy-dispersive X-ray (EDS) elemental mapping images were collected by HT-7700-TEM. The adsorption and desorption processes of the catalysts were analyzed by a specific surface area analyzer (Quanta chrome AUTOSORB-IQ2-MP) to determine the specific surface area. The pore size distribution was calculated from the adsorption isotherm data obtained via the nonlocal density functional theory (NLDFT) model. The electronic states of the catalyst surface were determined by X-ray photoelectron spectroscopy (XPS) (Thermo Scientific ESCALAB 250Xi, Al Ka1486.6 eV, He I 21.2 eV). Thermal stability was measured by thermal gravimetric analysis (TGA) Q50 under airflow of temperatures from 50 °C to 800 °C at a constant heating rate of 10 °C/min.

### 3.3. Preparation of HamZIF-90

First, 0.0096 g of melamine and 0.0105 g of salicylic acid were added to 20 mL of aqueous solution containing 0.05–0.08mol/L of zinc nitrate and stirred at 70 °C and 300 rpm for 15 min. Then 20 mL of hot Zn hydrogel aqueous solution was added to 80 mL of n-hexane containing 5.0 g of Span 85 and stirred at 60 °C and at 600 rpm for 1 h. Subsequently, the flask was transferred to an ice bath while stirring at 400 rpm for 30 min to promote agglutination gelation. Then, 1 mol/L of imidazole-2-carbaldehyde and 0.5 g of PVP were dissolved into 20 mL of water and stirred at 400 rpm for 1 h at 80 °C. Then it was mixed with the gel emulsion and continued to be stirred at 25 °C for 12 h. The MOFs containing hydrogels were collected by centrifugation and suspended in water at 70 °C for 6 h to remove the hydrogel template. Finally, HamZIF-90 was obtained by centrifugation, washed repeatedly with ethanol, and then dried under vacuum at 80 °C for 12 h before use.

### 3.4. Preparation of Ha_m_ZIF-90@Pd

The activated 100 mg of HamZIF-90 was dispersed in 20 mL of dichloromethane and sonicated for 30 min. The calculated Pd(acac)_2_ was dissolved in 0.5 mL of dichloromethane and then added to the Ha_m_ZIF-90 dispersion, sonicated for 30 min, and stirred for 24 h. Ha_m_ZIF-90 impregnated with Pd^2+^ was obtained by centrifugation and dried at 393 K for 6 h, and then HamZIF-90@Pd was obtained by reduction with 0.6 mol/L NaBH_4_ solutions.

### 3.5. Preparation of Ha_m_ZIF-90@Pd@CALB

Ha_m_ZIF-90@Pd (100 mg) was dispersed in 15 mL of isopropanol and sonicated for 5 min. Under stirring, 20 mg of CALB dispersed in 10 mL of phosphate buffer (0.1 M, pH = 7) was added to the suspension, and stirring was continued for 5 min, followed by the addition of 12 mL of glutaraldehyde solution (25 wt.%) and stirring overnight at 22 °C and 180 rpm in a shaker. The solid was separated by centrifugation, washed several times with ethanol, and then dried in a vacuum freeze dryer and prepared for use.

### 3.6. Dynamic Kinetic Resolution Reaction of 1-Phenylethylamine Catalyzed by Ha_m_ZIF-90@Pd@ CALB

The one-pot dynamic kinetic resolution reaction of 1-phenylethylamine was carried out in a Celec tube. The reaction system was as follows: 100 mg Ha_m_ZIF-90@Pd@CALB, 0.13 mmol 1-phenylethylamine, 0.15 mmol acyl donor, 4 mL solvent, 60 ℃, and a certain hydrogen pressure.

### 3.7. Quantitative Detection

The quantification of substrate and product was carried out by liquid chromatography (Fuli FL2200, Zhejiang Wenling Fuli Analytical Instruments Co., Ltd. (Wenling, China)) with a liquid chiral column CHIRALPAK IB N-5 (Daicel Chiral Technologies (China) Co., Ltd. (Shanghai, China), 250–4.6 mm column size). The mobile phase ratio was n-hexane/ethanol/ethanolamine = 90/10/0.1, the flow rate was 0.6 mL/min, the detection wavelength was 220 nm, and the injection was 5 μL. The enantiomeric excess of the product (ee_p_), the enantiomeric excess of the remaining substrate (ee_s_), and the conversion rate (conv.) were calculated as follows:

(1) $${\mathrm{ee}}_{S}=\frac{\left(c_{\mathrm{SS}}-c_{\mathrm{SR}}\right)}{\left(c_{\mathrm{SS}}{+c}_{\mathrm{SR}}\right)}\times 100\%$$

(2) $${\mathrm{ee}}_{P}=\frac{\left(c_{\mathrm{PR}}-c_{\mathrm{PS}}\right)}{\left(c_{\mathrm{PR}}{+c}_{\mathrm{PS}}\right)}\times 100\%$$

(3) $$\mathrm{Conv}.=\frac{{(c}_{\mathrm{PR}}{+c}_{\mathrm{BP}})}{\left(c_{\mathrm{PR}}{+c}_{\mathrm{BP}}{+c}_{S}\right)}\times 100\%$$

(4) $$\mathrm{Sel}._{R-\mathrm{amide}}=\frac{c_{\mathrm{PR}}}{{(c}_{\mathrm{PR}}{+c}_{\mathrm{BP}})}\times 100\%$$

where c_SR_, c_SS_, c_PR_, c_PS_, c_BP_, and c_S_ represent (R)-amine, (S)-amine, (R)-amide, (S)-amide, by-product, and substrate including (R)-amine and (S)-amine.

## 4. Conclusions

We prepared hollow amorphous MOFs using hydrogels as templates and constructed a combined catalyst Ha_m_ZIF-90@Pd@CALB that supported both chemical and biological catalysts and subsequently characterized them separately. The crystal diffraction peaks by XRD identified HamZIF-90 as amorphous, the energy spectra of XPS and EDS demonstrated the successful load of Pd NPs and CALB, the images of SEM and TEM indicated that it had a hollow structure, and the morphology size of HamZIF-90 was approximately 3 μm. At the same time, it was found that the cavity gradually became smaller with the increasing of zinc ion concentration, and we had carried out the DKR reaction by using 1-phenethylamine as the substrate and Ha_m_ZIF-90@Pd@CALB as the catalyst and obtained a maximum conversion of 98% and a selectivity of 93% for the reaction. Compared with ZIF-90@Pd@CALB, HamZIF-90@Pd@CALB exhibited better catalytic activity due to the hollow structure, which accelerated the molecular diffusion. Compared with HamZIF-90@Pd and Novozyme 435, the mass transfer distance was shortened due to the co-immobilization of Pd NPs and CALB, resulting in a better catalytic effect of HamZIF-90@Pd@CALB. It is worth stating that our work was focused on exploring the design of new MOFs that specifically support chemical and biological catalysts.

## Figures and Tables

**Table 1:** Racemization of *(S)*-1-phenylethylamine through Pd catalysts.

Entry	Catalyst	Conversion (%)	ee_amine_ (%)
1	Ha_m_ZIF-90(0.05)@Pd	33	12
2	Ha_m_ZIF-90(0.06)@Pd	42	12
3	Ha_m_ZIF-90(0.07)@Pd	38	17
4	Ha_m_ZIF-90(0.08)@Pd	35	13
5	Ha_m_ZIF-90(0.05)	/	/
6	Ha_m_ZIF-90(0.06)	/	/
7	Ha_m_ZIF-90(0.07)	/	/
8	Ha_m_ZIF-90(0.08)	/	/

Reaction conditions: Pd catalyst (75 mg), (*S*)-1-phenylethylamine (0.13 mmol), reaction time (12 h), P_H2_ (0.03 MPa), temperature (60 ℃), and toluene (4 mL).

**Table 2:** DKR reaction of (*rac*)-1-phenylethylamine.

Entry	Catalyst	Conversion (%)	ee_amine_ (%)	Sel._R-amide_ (%)
1	ZIF-90@Pd, Novozym 435	83	99	81
2	Ha_m_ZIF-90(0.05)@Pd, Novozym 435	78	99	74
3	Ha_m_ZIF-90(0.06)@Pd, Novozym 435	85	96	88
4	Ha_m_ZIF-90(0.07)@Pd, Novozym 435	86	97	84
5	Ha_m_ZIF-90(0.08)@Pd, Novozym 435	87	98	83
6	ZIF-90@Pd@CalB	75	99	73
7	Ha_m_ZIF-90(0.05)@Pd@CalB	82	99	78
8	Ha_m_ZIF-90(0.06)@Pd@CalB	98	99	93
9	Ha_m_ZIF-90(0.07)@Pd@CalB	91	99	76
10	Ha_m_ZIF-90(0.08)@Pd@CalB	89	99	80

Reaction conditions: Novozyme 435 (200 mg) and 4-Chlorophenyl-5-chloropentanoate (0.15 mmol); other conditions were the same as above.

**Table 3:** Influence of hydrogen pressure on DKR of 1-phenylethylamine.

Entry	Catalyst	P_H2_(MPa)	Conversion (%)	ee_p_ (%)	Sel._R-amide_ (%)
1	Ha_m_ZIF-90(0.06)@Pd@CalB	0.03	98	99	93
2	Ha_m_ZIF-90(0.06)@Pd@CalB	0.015	84	97	76
3	Ha_m_ZIF-90(0.06)@Pd@CalB	0.05	93	98	85

Reaction conditions: 75 mg catalyst, 0.13 mmol of *rac*-1-phenylethylamine, 0.15 mmol of 4-Chlorophenyl-5-chloropentanoate, reaction time 12 h, 4 mL toluene.

**Table 4:** Influence of solvents on the DKR of 1-phenylethylamine.

Entry	Catalyst	Solvent	Conversion (%)	ee_amine_ (%)	Sel._R-amide_ (%)
1	Ha_m_ZIF-90(0.06)@Pd@CalB	toluene	98	99	93
2	Ha_m_ZIF-90(0.06)@Pd@CalB	DMF	85	99	82
3	Ha_m_ZIF-90(0.06)@Pd@CalB	EA	81	99	69
4	Ha_m_ZIF-90(0.06)@Pd@CalB	EAC	89	99	80

Reaction conditions: 75 Pd catalyst, 0.13 mmol of *rac*-1-phenylethylamine, 0.15 mmol of 4-Chlorophenyl-5-chloropentanoate, reaction time 12 h, 60 ℃, and P _H2_ = 0.03 MPa.

## Data Availability

Not applicable.

## Sample Availability

Samples of the compounds are available from the authors.

## References

[1] Bartoszewicz A., Ahlsten N., Martin-Matute B. (2013). Enantioselective Synthesis of Alcohols and Amines by Iridium-Catalyzed Hydrogenation, Transfer Hydrogenation, and Related Processes. Chem.-Eur. J..

[2] Paetzold J., Backvall J.E. (2005). Chemoenzymatic dynamic kinetic resolution of primary amines. J. Am. Chem. Soc..

[3] Wang J.W., Li Y., Nie W., Chang Z., Yu Z.A., Zhao Y.F., Lu X., Fu Y. (2021). Catalytic asymmetric reductive hydroalkylation of enamides and enecarbamates to chiral aliphatic amines. Nat. Commun..

[4] Zhang X.M., Jing L.Y., Chang F.F., Chen S., Yang H.Q., Yang Q.H. (2017). Positional immobilization of Pd nanoparticles and enzymes in hierarchical yolk-shell@shell nanoreactors for tandem catalysis. Chem. Commun..

[5] Verho O., Bäckvall J.-E. (2015). Chemoenzymatic Dynamic Kinetic Resolution: A Powerful Tool for the Preparation of Enantiomerically Pure Alcohols and Amines. J. Am. Chem. Soc..

[6] Martín-Matute B., Bäckvall J.-E. (2007). Dynamic kinetic resolution catalyzed by enzymes and metals. Curr. Opin. Chem. Biol..

[7] Wang L.X., Wang L., Meng X.J., Xiao F.S. (2019). New Strategies for the Preparation of Sinter-Resistant Metal-Nanoparticle-Based Catalysts. Adv. Mater..

[8] Wang Y., Wang C., Wang L., Wang L., Xiao F.-S. (2021). Zeolite Fixed Metal Nanoparticles: New Perspective in Catalysis. Acc. Chem. Res..

[9] Yang L.-C., Deng H., Renata H. (2022). Recent Progress and Developments in Chemoenzymatic and Biocatalytic Dynamic Kinetic Resolution. Org. Process Res. Dev..

[10] Hermanová S., Zarevúcká M., Bouša D., Pumera M., Sofer Z. (2015). Graphene oxide immobilized enzymes show high thermal and solvent stability. Nanoscale.

[11] Li P., Moon S.-Y., Guelta M.A., Harvey S.P., Hupp J.T., Farha O.K. (2016). Encapsulation of a Nerve Agent Detoxifying Enzyme by a Mesoporous Zirconium Metal–Organic Framework Engenders Thermal and Long-Term Stability. J. Am. Chem. Soc..

[12] Aghababaie M., Beheshti M., Razmjou A., Bordbar A.-K. (2016). Covalent immobilization of Candida rugosa lipase on a novel functionalized Fe_3_O_4_@SiO_2_ dip-coated nanocomposite membrane. Food Bioprod. Process..

[13] Ferraz C.A., do Nascimento M.A., Almeida R.F.O., Sergio G.G., Junior A.A.T., Dalmonico G., Caraballo R., Finotelli P.V., Leao R.A.C., Wojcieszak R. (2020). Synthesis and characterization of a magnetic hybrid catalyst containing lipase and palladium and its application on the dynamic kinetic resolution of amines. Mol. Catal..

[14] Engstrom K., Johnston E.V., Verho O., Gustafson K.P.J., Shakeri M., Tai C.-W., Backvall J.-E. (2013). Co-immobilization of an enzyme and a metal into the compartments of mesoporous silica for cooperative tandem catalysis: An artificial metalloenzyme. Angew. Chem..

[15] Gustafson K.P.J., Lihammar R., Verho O., Engström K., Bäckvall J.-E. (2014). Chemoenzymatic Dynamic Kinetic Resolution of Primary Amines Using a Recyclable Palladium Nanoparticle Catalyst Together with Lipases. J. Org. Chem..

[16] Xu S., Wang M., Feng B., Han X., Lan Z., Gu H., Li H., Li H. (2018). Dynamic kinetic resolution of amines by using palladium nanoparticles confined inside the cages of amine-modified MIL-101 and lipase. J. Catal..

[17] Burtch N.C., Jasuja H., Walton K.S. (2014). Water Stability and Adsorption in Metal–Organic Frameworks. Chem. Rev..

[18] Benoit V., Chanut N., Pillai R.S., Benzaqui M., Beurroies I., Devautour-Vinot S., Serre C., Steunou N., Maurin G., Llewellyn P.L. (2018). A promising metal-organic framework (MOF), MIL-96(Al), for CO_2_ separation under humid conditions. J. Mater. Chem. A.

[19] Ding N., Li H.W., Feng X., Wang Q.Y., Wang S., Ma L., Zhou J.W., Wang B. (2016). Partitioning MOF-5 into Confined and Hydrophobic Compartments for Carbon Capture under Humid Conditions. J. Am. Chem. Soc..

[20] Qin L., Li Y., Liang F., Li L., Lan Y., Li Z., Lu X., Yang M., Ma D. (2022). A microporous 2D cobalt-based MOF with pyridyl sites and open metal sites for selective adsorption of CO_2_. Microporous Mesoporous Mater..

[21] Wang X., Li M., Cao C., Liu C., Liu J., Zhu Y., Zhang S., Song W. (2016). Surfactant-Free Palladium Nanoparticles Encapsulated in ZIF-8 Hollow Nanospheres for Size-Selective Catalysis in Liquid-Phase Solution. ChemCatChem.

[22] Ricco R., Wied P., Nidetzky B., Amenitsch H., Falcaro P. (2020). Magnetically responsive horseradish peroxidase@ZIF-8 for biocatalysis. Chem. Commun..

[23] Liang W.B., Xu H.S., Carraro F., Maddigan N.K., Li Q.W., Bell S.G., Huang D.M., Tarzia A., Solomon M.B., Amenitsch H. (2019). Enhanced Activity of Enzymes Encapsulated in Hydrophilic Metal-Organic Frameworks. J. Am. Chem. Soc..

[24] Qiu Y., Tan G., Fang Y., Liu S., Zhou Y., Kumar A., Trivedi M., Liu D., Liu J. (2021). Biomedical applications of metal–organic framework (MOF)-based nano-enzymes. New J. Chem..

[25] Qin L., Liang F., Li Y., Wu J., Guan S., Wu M., Xie S., Luo M., Ma D. (2022). A 2D Porous Zinc-Organic Framework Platform for Loading of 5-Fluorouracil. Inorganics.

[26] Nadar S.S., Vaidya L., Rathod V.K. (2020). Enzyme embedded metal organic framework (enzyme–MOF): De novo approaches for immobilization. Int. J. Biol. Macromol..

[27] Chen Y.W., Lv D.F., Wu J.L., Xiao J., Xi H.X., Xia Q.B., Li Z. (2017). A new MOF-505@GO composite with high selectivity for CO2/CH4 and CO2/N-2 separation. Chem. Eng. J..

[28] Wang M., Wang X., Feng B., Li Y., Han X., Lan Z., Gu H., Sun H., Shi M., Li H. (2019). Combining Pd nanoparticles on MOFs with cross-linked enzyme aggregates of lipase as powerful chemoenzymatic platform for one-pot dynamic kinetic resolution of amines. J. Catal..

[29] Stauch B., Fisher S.J., Cianci M. (2015). Open and closed states of Candida antarctica lipase B: Protonation and the mechanism of interfacial activation1. J. Lipid Res..

[30] Ortiz C., Ferreira M.L., Barbosa O., dos Santos J.C.S., Rodrigues R.C., Berenguer-Murcia Á., Briand L.E., Fernandez-Lafuente R. (2019). Novozym 435: The “perfect” lipase immobilized biocatalyst?. Catal. Sci. Technol..

[31] Park S.-C., Chang W.-J., Lee S.-M., Kim Y.-J., Koo Y.-M. (2005). Lipase-catalyzed transesterification in several reaction systems: An application of room temperature lonic liquids for bi-phasic production ofn-butyl acetate. Biotechnol. Bioprocess Eng..

[32] Cheng K.P., Svec F., Lv Y.Q., Tan T.W. (2019). Hierarchical Micro- and Mesoporous Zn-Based Metal-Organic Frameworks Templated by Hydrogels: Their Use for Enzyme Immobilization and Catalysis of Knoevenagel Reaction. Small.

[33] Zhan G.W., Zeng H.C. (2017). A Synthetic Protocol for Preparation of Binary Multi-shelled Hollow Spheres and Their Enhanced Oxidation Application. Chem. Mat..

[34] Cao S., Bennett T.D., Keen D.A., Goodwin A.L., Cheetham A.K. (2012). Amorphization of the prototypical zeolitic imidazolate framework ZIF-8 by ball-milling. Chem. Commun..

[35] Pan Y., Liu Y., Zeng G., Zhao L., Lai Z. (2011). Rapid synthesis of zeolitic imidazolate framework-8 (ZIF-8) nanocrystals in an aqueous system. Chem. Commun..

[36] Gholipoor O., Hosseini S.A. (2021). Phenol removal from wastewater by CWPO process over the Cu-MOF nanocatalyst: Process modeling by response surface methodology (RSM) and kinetic and isothermal studies. New J. Chem..

[37] Shieh F.-K., Wang S.-C., Yen C.-I., Wu C.-C., Dutta S., Chou L.-Y., Morabito J.V., Hu P., Hsu M.-H., Wu K.C.W. (2015). Imparting Functionality to Biocatalysts via Embedding Enzymes into Nanoporous Materials by a de Novo Approach: Size-Selective Sheltering of Catalase in Metal–Organic Framework Microcrystals. J. Am. Chem. Soc..

